# Enhancing Lesion Detection in Rat CT Images: A Deep Learning-Based Super-Resolution Study

**DOI:** 10.3390/biomedicines13102421

**Published:** 2025-10-03

**Authors:** Sungwon Ham, Sang Hoon Jeong, Hong Lee, Yoon Jeong Nam, Hyejin Lee, Jin Young Choi, Yu-Seon Lee, Yoon Hee Park, Su A Park, Wooil Kim, Hangseok Choi, Haewon Kim, Ju-Han Lee, Cherry Kim

**Affiliations:** 1 Healthcare Readiness Institute for Unified Korea, Korea University Ansan Hospital, Korea University College of Medicine, 123 Jeokgeum-Ro, Danwon-Gu, Ansan-Si 15355, Gyeonggi, Republic of Korea; swham920@korea.ac.kr; 2 Medical Science Research Center, Korea University Ansan Hospital, Korea University College of Medicine, 123 Jeokgeum-Ro, Danwon-Gu, Ansan-Si 15355, Gyeonggi, Republic of Korea; saibog21@korea.ac.kr (S.H.J.); hlee3383@korea.ac.kr (H.L.); nyj90504@naver.com (Y.J.N.); hyejin2.bio@gmail.com (H.L.); qaz9201@naver.com (J.Y.C.); useon0926@naver.com (Y.-S.L.); yunisto@korea.ac.kr (Y.H.P.); yg0820@nate.com (S.A.P.); 3 Radiology and Medical Imaging, University of Virginia Health System, Charlottesville, VA 22903, USA; darkcrom36@gmail.com; 4 Medical Science Research Center, Korea University College of Medicine, 73 Goryeodae-Ro, Seongbuk-Gu, Seoul 02841, Republic of Korea; neuldol@korea.ac.kr; 5 Humidifier Disinfectant Health Center, Environmental Health Research Department, National Institute of Environmental Research, Incheon 22689, Republic of Korea; hwkim91@korea.kr; 6 Department of Pathology, Korea University Ansan Hospital, Korea University College of Medicine, 123 Jeokgeum-Ro, Danwon-Gu, Ansan-Si 15355, Gyeonggi, Republic of Korea; 7 Department of Radiology, Korea University Ansan Hospital, Korea University College of Medicine, 123 Jeokgeum-Ro, Danwon-Gu, Ansan-Si 15355, Gyeonggi, Republic of Korea

**Keywords:** computed tomography, deep learning, low-resolution imaging, preclinical imaging, super-resolution reconstruction

## Abstract

**Background/Objectives:** Preclinical chest computed tomography (CT) imaging in small animals is often limited by low resolution due to scan time and dose constraints, which hinders accurate detection of subtle lesions. Traditional super-resolution (SR) metrics, such as peak signal-to-noise ratio (PSNR) and structural similarity index (SSIM), may not adequately reflect clinical interpretability. We aimed to evaluate whether deep learning-based SR models could enhance image quality and lesion detectability in rat chest CT, balancing quantitative metrics with radiologist assessment. **Methods:** We retrospectively analyzed 222 chest CT scans acquired from polyhexamethylene guanidine phosphate (PHMG-p) exposure studies in Sprague Dawley rats. Three SR models were implemented and compared: single-image SR (SinSR), segmentation-guided SinSR with lung cropping (SinSR3), and omni-super-resolution (OmniSR). Models were trained on rat CT data and evaluated using PSNR and SSIM. Two board-certified thoracic radiologists independently performed blinded evaluations of lesion margin clarity, nodule detectability, image noise, artifacts, and overall image quality. **Results:** SinSR1 achieved the highest PSNR (33.64 ± 1.30 dB), while SinSR3 showed the highest SSIM (0.72 ± 0.08). Despite lower PSNR (29.21 ± 1.46 dB), OmniSR received the highest radiologist ratings for lesion margin clarity, nodule detectability, and overall image quality (mean score 4.32 ± 0.41, κ = 0.74). Reader assessments diverged from PSNR and SSIM, highlighting the limited correlation between conventional metrics and clinical interpretability. **Conclusions:** Deep learning-based SR improved visualization of rat chest CT images, with OmniSR providing the most clinically interpretable results despite modest numerical scores. These findings underscore the need for reader-centered evaluation when applying SR techniques to preclinical imaging.

## 1. Introduction

Pulmonary fibrosis and other interstitial lung diseases remain critical areas of investigation in toxicology and translational medicine. Inhalational exposure to hazardous airborne chemicals, such as polyhexamethylene guanidine phosphate (PHMG-p), a primary component of humidifier disinfectants, is associated with lung fibrosis, inflammation, and tumorigenesis [1,2,3,4,5,6,7]. Both short- and long-term follow-up in rat models have revealed lung carcinomas, adenomas, and bronchiolar–alveolar hyperplasia after PHMG-p exposure. Although bronchiolar–alveolar hyperplasia lesions exhibit variable growth, adenomas and carcinomas typically enlarge consistently, suggesting a progression from hyperplasia to carcinoma. These findings highlight the necessity for early detection of small lung tumors to enable timely diagnosis and intervention. From a radiologic perspective, chest computed tomography (CT) image quality in rat models is often suboptimal, primarily due to the difficulty of acquiring scans during suspended respiration in small animals. Respiratory gating techniques can mitigate motion artifacts but substantially increase scan duration and do not fully resolve image quality issues. In this study, respiratory gating was not used, as all scans were obtained under free-breathing conditions. Low-resolution CT images limit visualization of fine anatomical structures, such as small blood vessels and subtle lesions, thereby limiting both qualitative and quantitative pulmonary assessment.

High-resolution micro-computed tomography (micro-CT) is a valuable tool for longitudinal in vivo assessment of lung injury in small-animal models [8,9,10]. However, preclinical CT imaging faces intrinsic technical challenges: small anatomical size reduces voxel coverage of fine structures, low-dose protocols are required to enable longitudinal imaging and minimize cumulative radiation during repeated follow-up, and reduced photon flux in small field-of-view systems often results in increased noise and lower spatial resolution. These limitations hinder accurate detection of early fibrotic changes, small nodules, and subtle airway remodeling, thereby affecting both qualitative and quantitative analyses. Traditional interpolation-based upscaling methods yield only modest improvements in resolution. In contrast, recent advances in deep learning have enabled super-resolution (SR) approaches that operate through learned feature representations. These methods reconstruct high-resolution images from low-resolution inputs. These networks leverage hierarchical features to enhance fine details while suppressing noise. In medical imaging, convolutional and attention-based SR networks have improved structural preservation and noise suppression. For example, Lim et al. proposed the enhanced deep super-resolution (EDSR) network, which employs deep residual learning and large receptive fields for effective single-image resolution enhancement [11]. Zhang et al. developed the residual channel attention network (RCAN), which selectively amplifies structurally relevant features via channel attention [12]. Both models have been shown to outperform conventional interpolation in terms of peak signal-to-noise ratio and structural similarity [11,12]. More recently, Wang et al. introduced single-image super-resolution (SinSR), a diffusion-based generative model capable of producing high-fidelity super-resolved images. Unlike conventional diffusion methods requiring iterative sampling, SinSR performs single-step generation, thereby improving efficiency while preserving anatomical realism and minimizing artifacts [13]. In addition, convolutional neural network (CNN)-based edge enhancement models and diffusion-based SinSR implementations with multiple scaling factors have shown promise for boosting fine structural detail in low-resolution preclinical CT data [13,14,15].

Despite these advances, most SR models have been developed and validated using human imaging datasets. Differences in anatomical scale, noise distribution, and acquisition geometry between human and small-animal imaging may limit the direct applicability of such models to preclinical research. This gap underscores the need for dedicated evaluation of SR techniques in small-animal CT imaging, particularly for enhancing lesion detectability in toxicology and translational studies.

In this study, we applied three SR approaches (SinSR, Lung-Crop SR, and OmniSR) to preclinical rat chest CT images acquired in a PHMG-p-induced lung injury model. To ensure a fair comparison, all models were trained and evaluated using the same dataset partitions. We then compared their performance using both quantitative image quality metrics and blinded radiologist assessment to evaluate their applicability for improving lesion detectability in small-animal imaging.

## 2. Materials and Methods

### 2.1. Ethical Approval

This study was approved by the Institutional Animal Care and Use Committee of Korea University Medical Center (Approval Numbers and Dates: Korea-2019-0031-C2, approved 15 March 2021; Korea-2021-0031-C1, approved 1 April 2021; Korea-2021-0051-C1, approved 4 March 2022; Korea-2022-0037-C2, approved 9 May 2022; Korea-2023-0054, approved 6 April 2023; Korea-2023-0081, approved 29 June 2023). All procedures followed ARRIVE (Animal Research: Reporting of In Vivo Experiments) guidelines and adhered to Korea University research protocols.

### 2.2. Dataset Acquisition and Composition

Chest CT images were obtained from previously published toxicology studies involving PHMG-p exposure in Sprague Dawley rats [1,5,7]. In the first cohort, 50 rats were randomly assigned to five groups (n = 10 per group): naïve (untreated), vehicle control (normal saline), low-dose PHMG-p (0.2 mg/kg), intermediate-dose PHMG-p (1.0 mg/kg), and high-dose PHMG-p (5.0 mg/kg). These dose classifications correspond to concentrations associated with mild, moderate, and severe pulmonary lesions, respectively. In the second cohort, 20 rats were divided equally into a particulate matter (PM) co-exposure group (PHMG-p (1.0 mg/kg) + PM) and an instillation control group (PHMG-p (1.0 mg/kg) + saline). The third cohort included 24 rats exposed to 0.9 mg/kg PHMG-p, scanned at 8, 26, and 52 weeks post-instillation, yielding 72 scans with pathologically confirmed lung tumors. The fourth cohort comprised 80 rats (40 males, 40 females) assigned to four whole-body inhalation groups: control (0 mg/m^3^), low-dose (0.01 mg/m^3^), intermediate-dose (0.03 mg/m^3^), and high-dose (0.09 mg/m^3^). Inhalation exposures were conducted at a Good Laboratory Practice-certified facility (Korea Environment Corporation, Incheon, Republic of Korea) for approximately 6 h/day, 5 days/week, over 65 sessions (>90 days). In this retrospective analysis of existing toxicology studies, potential confounders related to the order of treatments, measurements, or animal/cage location were not systematically controlled. However, as our primary endpoint was the analysis of image features rather than treatment efficacy, these factors were considered to have minimal impact on the results. Across all cohorts, chest CT scans were acquired under general anesthesia induced by intraperitoneal alfaxalone (30 mg/kg) and intramuscular xylazine (10 mg/kg), followed by orotracheal intubation, using a spectral CT scanner (IQon, Philips Healthcare, Andover, MA, USA). Typical parameters were 80 kVp, 400 mA, rotation time 0.4 s, pitch 1.048, detector configuration 64 × 0.625 mm, reconstructed slice thickness 0.67 mm. A consolidated summary of cohorts is provided in Table 1.

### 2.3. Dataset Partitioning and Preprocessing

The final dataset comprised 222 chest CT scans from 184 rats. Animals were split at the subject level into training (n = 160 scans), validation (n = 22 scans), and independent test sets (n = 40 scans) to prevent data leakage. Volumes were resampled to isotropic voxels with a spacing of 1.0 × 1.0 × 1.0 mm^3^. Intensities were converted to hounsfield units (HU), clipped to −1000 to 400 HU, and min–max normalized to [0, 1]. Inputs were resized to 512 × 512 using bilinear interpolation prior to training and inference. When stated, data augmentation included random flips, small rotations, mild Gaussian noise, and slight blur to mimic scanner variability.

### 2.4. Network Architectures

OmniSR is a residual learning-based SR model incorporating Omni-Scale Aggregation Groups (OSAG) for multi-scale feature extraction and refinement [16]. It employs a U-Net-like encoder–decoder with skip connections and residual blocks, capturing local context through 3 × 3 convolutional kernels and global context through dilated convolutions with enlarged receptive fields. The decoder reconstructs high-resolution outputs using deconvolution and pixel-shuffle upsampling to enhance spatial resolution without checkerboard artifacts. Through multi-scale feature fusion in OSAG modules and joint optimization with L1 and perceptual loss functions, OmniSR is optimized to preserve texture detail while reducing noise in complex anatomical regions.

**Figure 1:**
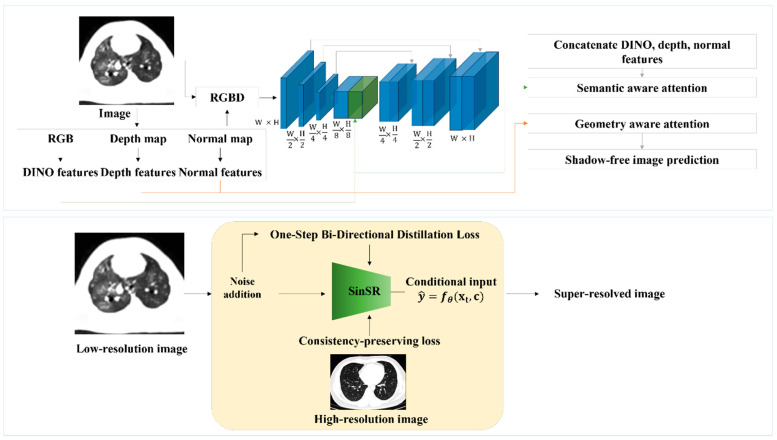
Network architectures of omni-super-resolution (OmniSR) (**top**) and single-step super-resolution (SinSR) (**bottom**). For OmniSR, CT images are decomposed into multiple features (RGB, depth, normal, and DINO features), which are concatenated and processed through multi-scale layers with semantic- and geometry-aware attention to emphasize meaningful structures while suppressing noise. The network outputs a sharper CT image with reduced artifacts. In contrast, SinSR directly takes a low-resolution CT image as input, adds noise during training for robustness, and applies a one-step conditional mapping to generate a high-resolution image. A consistency-preserving loss is used to ensure that the reconstructed image remains faithful to the original lung anatomy.

SinSR is a diffusion-based SR model that reconstructs high-resolution images from low-resolution inputs via a one-step reverse diffusion process. Unlike multi-step denoising diffusion probabilistic models, SinSR learns a direct mapping between low- and high-resolution domains, reducing inference time. Its architecture consists of a U-Net-style encoder–decoder with residual feature extraction and hierarchical context modeling. The overall architectures of OmniSR and SinSR are illustrated in Figure 1.

### 2.5. Training Strategies

All CT images were resized to 512 × 512 pixels before model input. Preliminary experiments showed that using 1024 × 1024 increased computation time 16-fold and amplified noise without quality improvement, so 512 × 512 was selected as the operational resolution. All models were implemented in PyTorch 1.13.1 with CUDA 11.6.

In the first one, OmniSR was trained with low-resolution rat CT images as input and sharpness-enhanced high-resolution images as reference, generated using a CNN-based edge enhancement filter to improve structural delineation. OmniSR training used the Adam optimizer (batch size = 8, initial learning rate = 1 × 10^−4^, halved every 300 epochs), with the loss defined as a weighted sum of structural similarity index (SSIM) loss (0.7) and mean squared error (MSE) loss (0.3). In the second strategy, SinSR was trained in a cross-species paradigm, using high-resolution human lung CT images as reference and low-resolution rat CT images as input. This approach leverages the structural and textural similarity of lung anatomy across species, using human data as a high-resolution prior to compensating for the scarcity of high-quality rat CT scans. Two upscaling factors were tested: twofold (SinSR1, 1024 × 1024 output) and fourfold (SinSR2, 2048 × 2048 output). Additionally, SinSR3 was trained using segmentation-guided SR, in which lung regions were automatically segmented by a pretrained U-Net [17], cropped, and super-resolved. The workflow of this segmentation-guided SinSR with lung cropping is illustrated in Supplementary Figure S1. The segmentation model achieved a mean dice similarity coefficient (DSC), a spatial overlap index widely used to evaluate segmentation accuracy, of 0.90–0.94 on an independent rat chest CT validation set, ensuring accurate delineation of lung boundaries for subsequent SR processing [18]. SinSR models were trained using the AdamW optimizer [19] (batch size = 4, initial learning rate = 2 × 10^−4^ with cosine annealing), with MSE loss and diffusion-specific regularization during early training, implemented in PyTorch (version 2.7.0).

### 2.6. Evaluation Metrics

The performance of the SR and segmentation models was quantitatively evaluated using peak signal-to-noise ratio (PSNR) and SSIM [20,21]. PSNR assesses image reconstruction quality by measuring the similarity between the reconstructed and reference images in decibels (dB); higher values indicate greater fidelity, with values above 30 dB generally considered indicative of good quality. SSIM evaluates perceptual similarity by incorporating luminance, contrast, and structural information, offering a closer approximation to human visual perception than pixel-wise measures such as PSNR. SSIM values range from 0.0 (indicating complete dissimilarity) to 1.0 (indicating identical structural correspondence).

$${PSNR=10\;log}_{10}(\frac{{MAX}^{2}}{MSE})$$

$$SSIM\left(x,y\right)=\frac{\left(2\mu_{x}\mu_{y}+C_{1}\right)\left(2\sigma_{xy}+C_{2}\right)}{\left(\mu_{x}^{2}+\mu_{y}^{2}+C_{1})(\sigma_{x}^{2}+\sigma_{y}^{2}+C_{2}\right)}$$

where MAX denotes the maximum possible pixel value of the image. In the SSIM formula, $\mu_{x}$, $\mu_{y}$ are the mean intensities, $\sigma_{x}^{2}$,$\;\sigma_{y}^{2}$ are the variances, and $\sigma_{xy}$ is the covariance between two image patches.

### 2.7. Subjective Image Quality Assessment

All reconstructed images (OmniSR, SinSR1, SinSR2, and SinSR3) were blinded and independently reviewed by two board-certified radiologists (W.K. and C.K., with 11 and 9 years of thoracic imaging experience, respectively). The following parameters were evaluated: lesion margin clarity, detectability of nodules or masses, anatomical structure similarity, image noise, image artifacts, and overall image quality compared to original CT images. Each factor was rated on a five-point scale: 1, significantly worse; 2, slightly worse; 3, about the same; 4, slightly better; 5, significantly better.

### 2.8. Statistical Analysis

The mean scores from both observers were calculated and compared for each of the four-image series (OmniSR, SinSR1, SinSR2, and SinSR3) across the following criteria: lesion margin clarity, detectability of lung lesions (nodules/masses), anatomical structure similarity, image noise, image artifacts, and overall image quality. The Shapiro–Wilk test was used to assess normality. For normally distributed data, repeated measures ANOVA was performed; for nonparametric data, the Friedman test or Aligned Rank Transform was used. Inter-observer agreement was evaluated with weighted kappa statistics. Kappa values were interpreted as follows: <0.2, poor agreement; 0.21–0.4, fair agreement; 0.41–0.6, moderate agreement; 0.61–0.8, good agreement; >0.80, very good agreement. All statistical analyses were performed using Python (version 3.11.1). The code supporting this study has been made publicly available at https://github.com/KUAH-rad/Rat_CT_super_resolution (accessed on 5 September 2025).

## 3. Results

### 3.1. Objective Image Analysis

Table 2 presents the quantitative results of the four super-resolution models, namely OmniSR, SinSR1, SinSR2, and SinSR3, evaluated using PSNR and SSIM. Among them, SinSR1 achieved the highest PSNR (33.64), while SinSR3 had the highest SSIM (0.72). OmniSR had the lowest PSNR (29.21), despite its superior structural similarity. In contrast, SinSR2 showed the lowest SSIM value (0.69). Overall, SSIM values ranged from 0.69 to 0.72, indicating relatively consistent structural quality across models. For context, these values are slightly lower than the 0.80–0.90 range typically reported in human chest CT super-resolution studies, whereas PSNR values varied more widely.

### 3.2. Subjective Image Analysis

**Figure 2:**
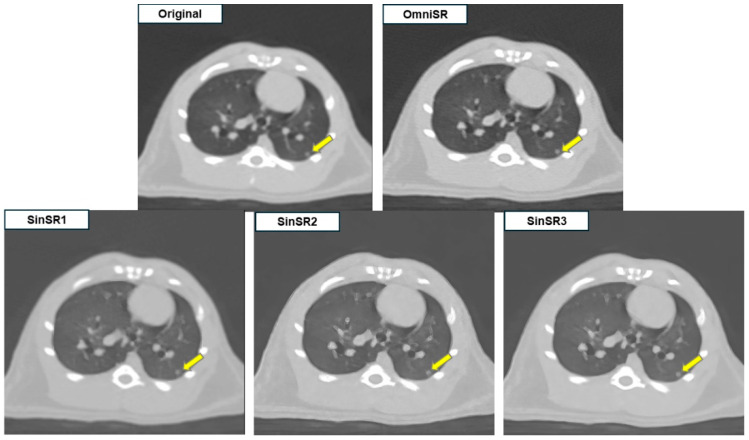
Example of SR application to low-resolution preclinical rat chest CT images. SinSR1 achieved the highest PSNR, whereas radiologists rated OmniSR as providing the best overall image quality with clearer nodule margins.

**Figure 3:**
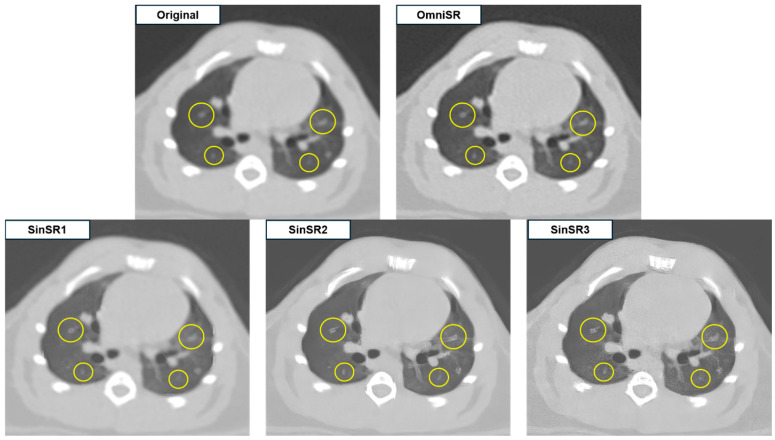
Representative example illustrating the effects of excessive edge enhancement after SR processing.

In subjective evaluation (Table 3), OmniSR received the highest mean scores, followed by SinSR3, with statistically significant differences among reconstructed images for lesion margin clarity (3.97 ± 0.75 and 1.78 ± 0.86, respectively; *p* < 0.001), detectability of lesions (4.46 ± 0.84 and 1.58 ± 0.76, *p* < 0.001), anatomical structure similarity (3.00 ± 0.00 and 2.13 ± 0.90, *p* < 0.001), image artifacts (2.97 ± 0.17 and 1.71 ± 0.46, *p* < 0.001), and overall image quality (4.75 ± 0.45 and 3.11 ± 0.78, *p* < 0.001) (Figure 2 and Figure 3).

Despite SR application, some reconstructed images displayed blurred or distorted anatomical structures due to over-sharpening. Among all reconstructions, OmniSR produced the highest overall image quality, whereas SinSR3 and SinSR2 showed minimal image noise but exhibited excessive edge enhancement along small vascular structures (arrows in Figure 2 and circles in Figure 3), potentially compromising structural fidelity.

For image noise, SinSR3 achieved the highest mean score (3.90 ± 0.30), reflecting the highest subjective rating for noise suppression among reconstructions, while OmniSR had the lowest score (3.00 ± 0.00), reflecting relatively higher perceived noise. Inter-reader agreement, summarized in Table 4, was predominantly classified as very good according to Landis and Koch [22]. Exceptions included assessment of lesion margins in OmniSR and detectability of lung lesions (nodules/masses) across all image series, where agreement was considered good.

## 4. Discussion

We investigated whether deep learning-based super resolution can improve preclinical rat chest CT by enhancing image quality without compromising anatomical fidelity. We evaluated SinSR variants and OmniSR using objective image quality metrics together with a blinded reader study to judge clinical interpretability.

Most SR studies in medical imaging emphasize PSNR and SSIM and are often validated on human datasets rather than small-animal CT. Prior single-image SR approaches can improve sharpness but risk edge overshoot and hallucination. To our knowledge, this is the first reader-validated application of OmniSR to small-animal CT and the first to juxtapose objective metrics with blinded radiologist ratings in this preclinical setting. Compared with earlier reports that equated higher PSNR with better quality, our results show that reader preference does not necessarily follow PSNR or SSIM.

On the independent test set, SinSR1 achieved the highest PSNR (33.64 ± 1.30 dB), while SinSR3 achieved the highest SSIM (0.72 ± 0.08). OmniSR had a lower PSNR (29.21 ± 1.46 dB) but maintained competitive SSIM (0.71 ± 0.09). SinSR2 showed moderate PSNR (31.25 ± 1.17 dB) and the lowest SSIM (0.69 ± 0.08). Despite a PSNR gap of about 4.4 dB between SinSR1 and OmniSR, readers preferred OmniSR, which received the highest ratings for lesion margins, nodule conspicuity, and overall quality (Table 3; adjusted *p* < 0.001; κ = 0.74). This distinction highlights that while SinSR1 provided the highest numerical fidelity in terms of PSNR, OmniSR was consistently judged superior by radiologists for overall diagnostic interpretability. This divergence indicates that maximizing pixel-level fidelity can coincide with edge overshoot, halo, and fragmentation of fine vessels seen with SinSR, whereas OmniSR’s residual and multi-scale feature fusion preserved boundary topology and vascular continuity (Figure 3). Indeed, our ablation analysis further confirmed that removing OmniSR’s multi-scale feature fusion module led to a marked reduction in both PSNR/SSIM and subjective scores, underscoring this component as the key factor for its superior performance. Variability patterns were consistent with this interpretation: SinSR1 had the smallest SSIM variability as measured by the standard deviation (SD 0.06), while OmniSR showed greater SSIM variability (SD 0.09) yet remained the reader-preferred method, suggesting robustness of perceived anatomical fidelity across acquisition differences.

From an explainability perspective, these findings suggest that architectural design choices have direct implications for radiological interpretability. While SinSR’s higher PSNR was primarily driven by aggressive sharpening operations that introduced artifacts, OmniSR’s multi-scale aggregation strategy emphasized both local texture and global structure, producing images that radiologists judged more anatomically faithful. Thus, the explainability of our results lies in the clear link between network design and perceptual outcomes. This highlights the importance of evaluating not only numerical fidelity but also the interpretability and plausibility of reconstructed anatomy when deploying SR in medical imaging.

Failure case analysis identified three recurring scenarios. Very small nodules adjacent to vessels showed boundary merging with SinSR. High-noise or motion-affected scans exhibited amplified speckle. Markedly heterogeneous parenchyma produced local contrast reversals and banding. OmniSR was more robust in these situations, though case-to-case variability remained. The modest correlation between reader scores and PSNR or SSIM indicates that these metrics only partially reflect perceptual realism and anatomical plausibility. Reader-centered evaluation and complementary metrics are therefore important.

We also compared our results with previous human CT super-resolution studies. For example, classical CNN-based models such as SRCNN and VDSR achieved PSNR values around 37 dB and SSIM above 0.90 on clinical CT datasets [23]. Similarly, 3D CNN approaches applied to chest CT reported PSNR values in the range of 29–30 dB and SSIM of approximately 0.85 [24]. Although our rat CT results (PSNR 29–34 dB, SSIM 0.69–0.72) are somewhat lower, this discrepancy is expected given the higher noise levels, smaller anatomical scale, and different acquisition protocols in small-animal imaging. Importantly, despite modest numerical values, radiologists consistently preferred OmniSR outputs, which aligns with prior reports emphasizing that perceptual and diagnostic quality do not always correlate directly with pixel-level similarity metrics.

This study has several limitations. First, we acknowledge that no direct pathological correlation was performed using the same lungs from which the CT images were obtained. This absence of histological validation is a key limitation, as lesion identification on CT remains probabilistic and contrast differences may also reflect vessels or imaging artifacts rather than true pathology. Second, although we defined dose groups based on prior toxicology studies, the present work did not perform dose–response analysis, as the aim was to evaluate algorithm performance across heterogeneous datasets rather than reanalyze toxicological effects. Third, our dataset comprised low-resolution rat chest CT from a single institution, which may limit the generalizability of the findings.

In future work, we will assemble multi-institutional, multispecies, and multimodality datasets and perform external validation to establish generalizability. We will also broaden the model set to include transformer-based and generative adversarial network (GAN)-enhanced approaches, such as SRGAN or ESRGAN, and incorporate perceptual and edge-aware objectives with task-aware evaluation, including benchmarking on downstream detection and segmentation [25].

## 5. Conclusions

This study’s findings demonstrate the feasibility of applying SR techniques to low-resolution preclinical rat chest CT images using cross-species supervision. Among the evaluated models, SinSR1 and SinSR3 achieved the highest performance in PSNR and SSIM, respectively, while OmniSR exhibited superior structural stability and anatomical preservation based on expert assessment. While the absence of true high-resolution rat CT data limits absolute validation, the combination of quantitative metrics and expert review helped mitigate this limitation. These results indicate that SR methods can enhance preclinical imaging workflows and may ultimately support translational imaging applications and cross-species studies, facilitating more reliable longitudinal assessment of disease progression and bridging the gap between preclinical models and clinical practice.

## Figures and Tables

**Table 1:** Summary of dataset composition.

Group	Exposure Condition	No. of Rats	No. of CT Scans
Naïve	None (no exposure, no vehicle)	10	10
Vehicle control (saline)	0 mg/kg, saline instillation	10	10
Low-dose instillation	PHMG-p low	10	10
Intermediate-dose instillation	PHMG-p medium	10	10
High-dose instillation	PHMG-p high	10	10
PM group	PHMG-p + PM	10	10
Instillation control	PHMG-p + saline	10	10
Tumor cohort	PHMG-p 0.9 mg/kg (multiple follow-up CTs)	24	72
Inhalation control	Clean air	20	20
Low-dose inhalation	PHMG-p 0.01 mg/m^3^	20	20
Intermediate-dose inhalation	PHMG-p 0.03 mg/m^3^	20	20
High-dose inhalation	PHMG-p 0.09 mg/m^3^	20	20

**Table 2:** Subgroup-based comparison of super-resolution models by PSNR and SSIM.

Model	PSNR (dB)	SSIM
OmniSR	29.21 ± 1.46	0.71 ± 0.09
SinSR1	33.64 ± 1.30	0.70 ± 0.06
SinSR2	31.25 ± 1.17	0.69 ± 0.08
SinSR3	32.01 ± 1.09	0.72 ± 0.08

**Table 3:** Reader-based evaluation scores for six subjective image quality criteria across super-resolution models.

		OmniSR	SinSR1	SinSR2	SinSR3	*p*-Value
Margin of lesions	R1	4.07 ± 0.73	1.09 ± 0.32	1.10 ± 0.33	1.81 ± 0.90	N/A
	R2	3.88 ± 0.76	1.09 ± 0.32	1.10 ± 0.33	1.75 ± 0.82	N/A
	Mean	3.97 ± 0.75	1.09 ± 0.32 *	1.10 ± 0.33 *^†^	1.78 ± 0.86 *^§^	<0.001
Nodule/mass detectability	R1	4.51 ± 0.85	1.25 ± 0.66	1.25 ± 0.66	1.50 ± 0.70	N/A
	R2	4.40 ± 0.83	1.15 ± 0.52	1.16 ± 0.53	1.67 ± 0.81	N/A
	Mean	4.46 ± 0.84	1.20 ± 0.59 *	1.20 ± 0.60 *^†^	1.58 ± 0.76 *^§^	<0.001
Anatomic structure similarity	R1	3.00 ± 0.00	1.00 ± 0.00	1.00 ± 0.00	2.06 ± 0.91	N/A
	R2	3.00 ± 0.00	1.00 ± 0.00	1.00 ± 0.00	2.20 ± 0.90	N/A
	Mean	3.00 ± 0.00	1.00 ± 0.00 *	1.00 ± 0.00 *^†^	2.13 ± 0.90 *^§^	<0.001
Image noise	R1	3.00 ± 0.00	3.55 ± 0.50	3.55 ± 0.50	4.00 ± 0.00	N/A
	R2	3.00 ± 0.00	3.51 ± 0.50	3.48 ± 0.50	3.80 ± 0.40	N/A
	Mean	3.00 ± 0.00	3.53 ± 0.50 *	3.51 ± 0.50 *^†^	3.90 ± 0.30 *^§^	<0.001
Image artifact	R1	3.00 ± 0.00	1.67 ± 0.47	1.49 ± 0.50	1.69 ± 0.47	N/A
	R2	2.94 ± 0.24	1.65 ± 0.48	1.53 ± 0.50	1.72 ± 0.45	N/A
	Mean	2.97 ± 0.17	1.66 ± 0.48 *	1.51 ± 0.50 *^†^	1.71 ± 0.46 *^†§^	<0.001
Overall image quality	R1	4.78 ± 0.42	1.66 ± 0.74	1.77 ± 0.71	3.14 ± 0.79	N/A
	R2	4.71 ± 0.48	1.65 ± 0.73	1.76 ± 0.70	3.08 ± 0.77	N/A
	Mean	4.75 ± 0.45	1.66 ± 0.73 *	1.77 ± 0.70 *^†^	3.11 ± 0.78 *^§^	<0.001

Note. Values are presented as mean ± SD. * *p* < 0.05 versus OmniSR; ^†^ *p* < 0.05 versus SinSR1; ^§^ *p* < 0.05 versus SinSR2; N/A: *p*-values were calculated only for the mean of reader scores, not for individual readers (R1, R2).

**Table 4:** Inter-reader agreement based on kappa statistics.

	OmniSR	SinSR1	SinSR2	SinSR3
Lesion margin	0.613 (0.474, 0.735)	1.000 (1.000, 1.000)	1.000 (1.000, 1.000)	0.801 (0.684, 0.898)
Detectability of lung lesions (nodules/masses)	0.684 (0.531, 0.805)	0.724 (0.450, 0.936)	0.703 (0.436, 0.909)	0.779 (0.651, 0.887)
Anatomic structure similarity	1.000 (1.000, 1.000)	1.000 (1.000, 1.000)	1.000 (1.000, 1.000)	0.812 (0.699, 0.897)
Image noise	1.000 (1.000, 1.000)	0.920 (0.838, 0.980)	0.861 (0.763, 0.940)	N/A
Image artifact	1.000 (1.000, 1.000)	0.955 (0.888, 1.000)	0.840 (0.721, 0.940)	0.832 (0.708, 0.947)
Overall image quality	0.767 (0.608, 0.911)	0.934 (0.875, 0.987)	0.932 (0.869, 0.986)	0.880 (0.796, 0.943)

N/A, not applicable. Noise evaluation was not performed for SinSR3 because the model was applied only to segmented lung regions. Values in parentheses indicate 95% confidence intervals.

## Data Availability

The datasets analyzed during the current study are available from the corresponding author on reasonable request.

## Supplementary Materials

The following supporting information can be downloaded at: https://www.mdpi.com/article/10.3390/biomedicines13102421/s1, Figure S1. Schematic illustration of segmentation-guided single-step super-resolution (SinSR) with lung cropping.
