# Supplementary material for: Enhancing Lesion Detection in Rat CT Images: A Deep Learning-Based Super-Resolution Study
Source: Biomedicines. 2025 Oct 3;13(10):2421. doi: 10.3390/biomedicines13102421 (PMC12561881; doi:10.3390/biomedicines13102421)
Supplement: Supplementary file 1 [file biomedicines-13-02421-s001.zip › biomedicines-3839913-supplementary.pdf]

Supplementary materials

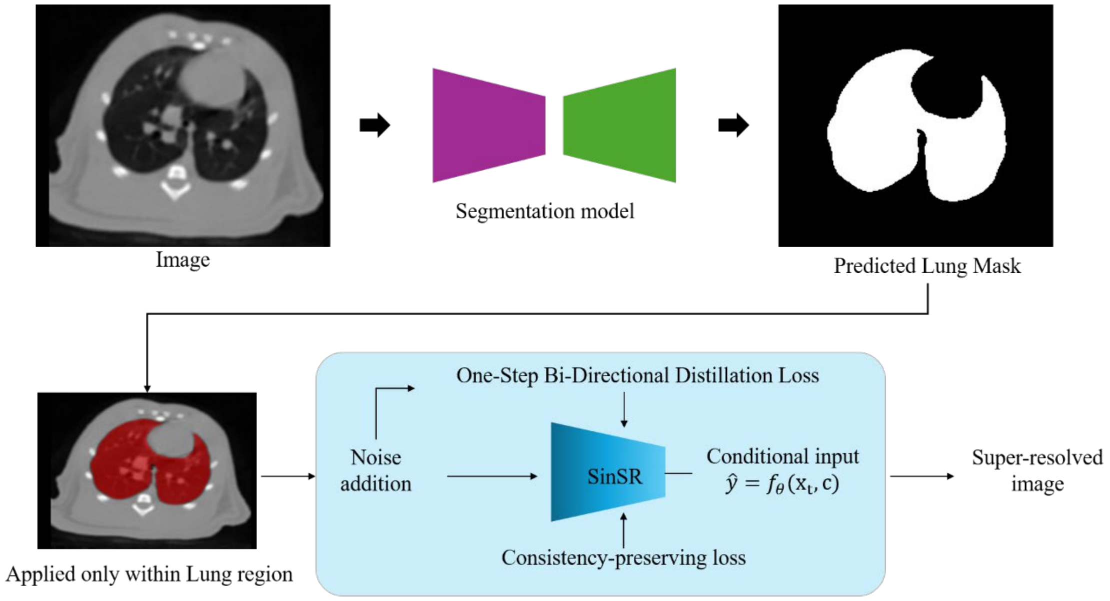

Figure S1. Schematic illustration of segmentation-guided single-step super-resolution (SinSR) with lung cropping.

Workflow of the proposed segmentation-guided SinSR with lung cropping (Lung-Crop SR). A chest CT slice was processed by a U-Net-based segmentation network to generate lung masks. The lung region was cropped and used as conditional input to the SinSR model, thereby restricting super-resolution to the lung parenchyma.
